# Autosomal Recessive Polycystic Kidney Disease: The Complete Molecular Architecture for the *PKHD1* Gene and Its Protein

**DOI:** 10.34133/csbj.0261

**Published:** 2026-11-12

**Authors:** Joshua P. Pillai, Sidhant N. Puntambekar, Ana C. Onuchic-Whitford, Frederico Ferreira, Matthew G. Sampson, Luiz F. Onuchic, John A. Sayer

**Affiliations:** ^1^ Division of Nephrology, Boston Children’s Hospital, Boston, MA, USA.; ^2^ Department of Pediatrics, Harvard Medical School, Boston, MA, USA.; ^3^ Kidney Disease Initiative, Broad Institute, Cambridge, MA, USA.; ^4^ Division of Renal Medicine, Brigham and Women’s Hospital, Boston, MA, USA.; ^5^ Division of Nephrology, School of Medicine, University of São Paulo, São Paulo, Brazil.; ^6^ Division of Molecular Medicine, School of Medicine, University of São Paulo, São Paulo, Brazil.; ^7^ Newcastle University, Newcastle Upon Tyne, UK.; ^8^ Newcastle Hospitals NHS Foundation Trust, Newcastle Upon Tyne, UK.; ^9^ NIHR Newcastle Biomedical Research Centre, Newcastle Upon Tyne, UK.

## Abstract

Fibrocystin/polyductin is a large single-pass transmembrane glycoprotein involved in tubulogenesis and maintenance of the epithelium, where disease-causing variants in its encoding gene *PKHD1* cause autosomal recessive polycystic kidney disease. While enormous experimental efforts have been taken to characterize proteins involved in autosomal dominant polycystic kidney disease, the structural and molecular basis of fibrocystin has lagged behind with no known mechanistic models. Here, our study introduces the first idealized molecular model of fibrocystin, where we confirm the established domain architecture and refine the nomenclature of unresolved regions. Using our molecular model, we show that disease-causing missense variants tend to be buried and impose marked loss-of-function effects to the monomer. Furthermore, our molecular model suggests that the protective kidney survival outcomes associated with missense variants from amino acids 709–1837 are likely a result of their surface exposure on the immunoglobulin-like plexin-transcription factor domains. Likewise, the protective hepatic outcomes of missense variants occurring in amino acids 1838–2624 are predicted to impose weakly destabilizing effects compared to all other functional classes of variants. Overall, our idealized model is the first to provide potential hypotheses addressing the broad diversity of phenotypes, thereby laying a foundation for basic and translational efforts for autosomal recessive polycystic kidney disease.

## Introduction

Autosomal recessive polycystic kidney disease (ARPKD) is an inherited hepatorenal disorder with an estimated incidence of 1 in 20,000 live births [1]. This disease is characterized by cystic dilations of the kidney collecting ducts and developmental anomalies of the hepatobiliary ductal plate, which lead to congenital hepatic fibrosis of heterogeneous severity. Such defects typically translate into enlarged cystic kidneys with progressive kidney disease, cystic/dilated intrahepatic bile ducts associated with hepatic fibrosis (Caroli syndrome), and, very occasionally, pancreatic cysts [2]. Nearly half of the patients with ARPKD that survive the neonatal period progress to kidney failure within the 2 first decades of life, requiring dialysis or kidney transplantation [3]. More than 90% of ARPKD cases are caused by biallelic disease-causing variants in *PKHD1* (PKHD1 Ciliary IPT Domain Containing Fibrocystin/Polyductin gene NM_138694.4) encoding the protein fibrocystin or polyductin [4–6]. Fibrocystin is a single-pass transmembrane glycoprotein that is involved in cellular adhesion, ciliary function, and cell proliferation in the kidney, but its functional roles and mechanisms remain poorly understood [7].

Fibrocystin is composed of 4,074 amino acids and has an estimated mass of 447 kDa based on its amino acid (AA) sequence, with no resolved experimental structures reported in current literature. However, biophysical analyses have identified structural motifs within the ectodomain, including 12 immunoglobulin-like plexin-transcription factor (IPT) domains, a protective antigen 14 (PA14) domain, 2 regions of CEMPI2 homology each with multiple parallel beta-helix 1 (PbH1) repeats, and a sea urchin sperm protein, enterokinase and agrin (SEA) domain [7]. Defining the structure of fibrocystin is essential for uncovering its role as a potential cell surface receptor, its involvement in protein–carbohydrate interactions, and the mechanisms by which proteolysis regulates its signaling. These efforts may expand a platform of knowledge potentially applicable to therapeutic aims, particularly given the absence of approved treatments for ARPKD.

Unfortunately, cryogenic electron microscopy (cryo-EM) and nuclear magnetic resonance (NMR) methods are currently unfeasible for resolution of the fibrocystin structure due to its molecular size and large ectodomain. A similar issue occurs for the PKD1–PKD2 complex, where cryo-EM failed to resolve the intracellular PLAT domain and all of the N-terminal region of PKD1 [8]. The complete global structure of the N-terminal PKD1 region cannot be resolved and is limited to resolution of isolated domains as observed with the PKD1 module [9]. Furthermore, murine models of *PKHD1* are preliminary with simple knockouts and lacking sufficient biochemical data of particular founder alleles [10–14]. Lastly, deep mutational scanning experiments of *PKHD1* are unfeasible due to molecular size limitations [15]. Overall, with limited developments for *in vivo* murine models, biophysical models, and *in vitro* minigene functional assays for the last 2 decades, there are currently substantial roadblocks to improving diagnostics, variant-clinical correlations, and therapeutic developments for ARPKD.

Thus, the genotype–phenotype correlations remain primitive and exploratory for ARPKD. It has been suggested that missense variants in AA709–1837 are protective against kidney failure without any mechanistic insights [16]. Missense variants in AA1838–2624 have also been claimed to cause a protective phenotype for portal hypertension and liver complications [16]. These correlations should be interpreted cautiously as Bonferroni correction of all kidney survival outcomes demonstrate null results across fibrocystin functional classes (Table S1).

Following the establishment of the essential architecture of fibrocystin [5], including the IPT and PbH1 domains, this study sought to establish the nomenclature for the remaining unsolved regions, predict the molecular basis for essential *PKHD1* variants, and provide a hypothesis for the phenotypes of the kidney and hepatic outcomes among *PKHD1* missense functional classes.

## Results

### A predicted molecular basis for the established domain architecture of fibrocystin

Since the identification of *PKHD1* by Onuchic *et al*. [5], its established architecture includes the PA14, IPT 1–12, G8, PbH1, and the SEA domain. At the C-terminal region, there is a transmembrane domain, ciliary targeting sequence, mitochondrial targeting sequence, and a nuclear localization sequence. In Fig. 1A, we provide an illustration of the known molecular architecture of fibrocystin and include the residue coordinates in Table 1. Although we have predicted the secondary structures of these domains based on sequence, we wanted to visualize their predicted molecular basis and confirm their broad folding patterns using AlphaFold 3 (AF3) [17]. Internal validation metrics (Table S2) of the predicted structure indicate that it is highly confident with a predicted local distance difference test (pLDDT) score of 73.59 (Fig. 1B). From this monomeric template, we labeled the established domains (Fig. 1C) and compared their prediction against known homologs.

**Fig. 1.**
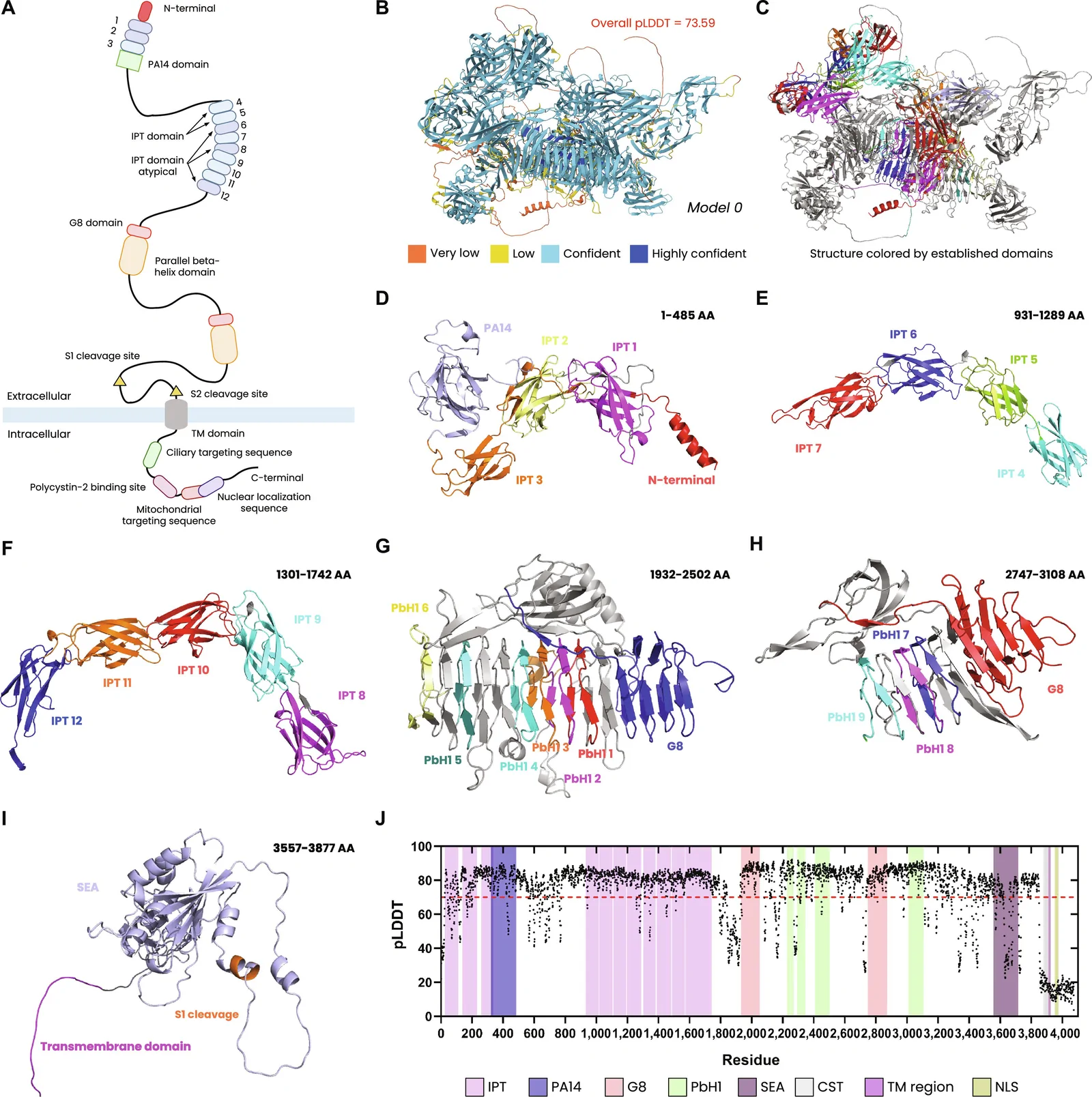
Domain architecture of fibrocystin from AlphaFold 3 (AF3) model. (A) Schematics for key features and domain architecture of fibrocystin, inspired by Bannell and Cockburn [7]. (B) AF3 prediction of fibrocystin, colored by predicted local distance difference test (pLDDT) scores with an overall score of 73.59, indicating a confident predicted model. (C) The AF3 fibrocystin model has been colored by its predicted domains obtained from UniProt (P08F94). The fibrocystin model has been isolated to visualize the (D) N-terminal to PA14 domains, (E) IPT 4 to IPT 7 domains, (F) IPT 8 to IPT 12 domains, (G) G8 1 to PbH1 6 domains, (H) G8 2 to PbH1 9 domains, and (I) SEA domain containing the S1 cleavage site before the transmembrane domain. (J) The local pLDDT for all residues has been visualized to identify domains of high confidence from AF3. The pLDDT per domain remains relatively greater than the threshold of 70. Generally, the pLDDT scores among residues between 2 domains tend to have poor predictions, including the C-terminal region. For IPT 1–2, they are partially of lower confidence due to the marked number of loops involved in the structure, but the remaining IPT 3 to IPT 12 are of high confidence. Due to the highly accurate prediction of most predicted domains, localized analysis of the structure was enabled. AA, amino acid; CTS, ciliary targeting sequence; PA14, protective antigen 14; IPT, immunoglobulin-like plexin-transcription factor; PbH1, parallel beta-helix 1; SEA, sea urchin sperm protein, enterokinase, and agrin; TM, transmembrane.

**Table 1.** Protein domains of known molecular architecture of fibrocystin, AlphaFold confidence scores (predicted local distance difference test, pLDDT), and residue coordinates

Protein domain	Mean pLDDT	Residues
IPT 1	67.16244	24–111
IPT 2	74.92523	137–232
IPT 3	80.14056	259–335
PA14	76.46314	322–485
IPT 4	78.13655	931–1015
IPT 5	77.28815	1019–1103
IPT 6	78.81182	1108–1192
IPT 7	73.58243	1196–1289
IPT 8	72.73732	1301–1382
IPT 9	75.59924	1389–1481
IPT 10	75.32618	1486–1570
IPT 11	79.27985	1573–1659
IPT 12	77.71937	1658–1742
G8 1	80.6499	1932–2053
PbH1 1	81.93161	2226–2248
PbH1 2	82.22006	2249–2271
PbH1 3	76.90404	2292–2325
PbH1 4	83.09054	2326–2347
PbH1 5	81.39089	2409–2431
PbH1 6	81.15746	2469–2502
G8 2	77.66285	2747–2873
PbH1 7	83.08782	3010–3032
PbH1 8	83.71332	3033–3055
PbH1 9	82.05948	3086–3108
SEA	60.64556	3557–3717
TM	30.27444	3859–3881
CST	28.51502	3876–3893
NLS	24.44438	3952–3976

CST, ciliary targeting sequence; IPT, immunoglobulin-like plexin-transcription factor; NLS, nuclear localization sequence; SEA, sea urchin sperm protein, enterokinase, and agrin; TM, transmembrane

We confirm that the PA14 domain forms a globular β-barrel composed of 9 β-strands and the IPT domains form immunoglobulin-like folding patterns (Fig. 1D). The IPT domains impart a curved ectodomain to fibrocystin, observed in homologs (Protein Data Bank: 5L59), that has been suggested to be involved in dimerization and signaling. The curvature continues throughout all IPT domains (Fig. 1D to F), with substantially greater curving either contributed from the flexible intrinsically disordered connective loops or the native structure in IPT 9–12.

As reported in the discovery of the G8 domain [18], we confirm the domain is composed of 10 β-strands and 1 α-helix, where 9 β-strands are directly involved in formation of a head with a β-helical trunk and the last strand is connected separately with the interdomain leading to the first PbH1 domain (Fig. 1G). From both Mayans *et al*. [19] and homologs (Protein Data Bank: 8C6I), we confirm that each PbH1 repeat is composed of 3 β-strands, stacking into 6 total repeats. The 2 stacked β-sheets separating PbH1 4 from PbH1 5 are composed of 3 wound β-strands. Likewise, the single wound sheet separating PbH1 5 from PbH1 6 is composed of 2 β-strands (Fig. 1G). Similarly to G8 1, we identified 10 β-strands and 1 α-helix of G8 2, and 3 β-strands stacking to form PbH1 7 and 8 (Fig. 1F). However, we only identified 2 β-strands being stacked for PbH1 8, and the remaining sequence appeared as disordered loops (Fig. 1F).

Furthermore, prior experimental efforts validated the SEA domain as the S1 cleavage domain [20], where the cleavage sequence (“KRKR” motif) occurs on an α-helix connected between 2 disordered loops as found through conservation analysis (Fig. 1I). Naturally, the ciliary targeting sequence, mitochondrial targeting sequence, nuclear localization sequence, and C-terminal were all disordered. However, owing to low confidence in the region, the transmembrane helix failed to be predicted. In Fig. 1J, we included the pLDDT scores for all residues of fibrocystin and highlighted the local confidence of each domain in Table 1.

This biophysical analysis refines the established domain architecture for fibrocystin. Cryo-EM or NMR resolution structural studies are awaited for true validation, but we now have a reasonable framework for evaluating *PKHD1* missense variants. This study emulates the analyses by the International Polycystic Kidney Disease Consortium in defining the architecture for PKD1, but for fibrocystin [21].

### Discovery of the C6 cassette, 6 Glu-binding motifs, and the PbH1 10/11 repeat domains of fibrocystin

Next, our objective was to resolve the nomenclature for the novel unresolved domains, which would complete the long-awaited architecture of fibrocystin. Our study design followed a similar approach to those used to establish the G8 domain [18].

We first analyzed the N-terminal interdomains in the IPT 1–3, PA14, and IPT 4–6, which appear to be part of the galactose-binding domain-like superfamily (*E* = 6.19 × 10^−26^). With our structural framework, we narrowed it to 3 distinct units including residues 487–497 and 786–885, 547–722, and 498–546 and 723–785 (Fig. 2A to C). The region 547–722 is the most promising candidate as a functional domain owing to its noteworthy folding patterns. Therefore, we searched for homologs using HMMER [22], where we identified 699 homologs. We reduced the possible homologs to include those that are not fibrocystin isoforms or fibrocystin-L (*N* = 36), which we further reduced to include PA14 domains upstream (*N* = 19) (Fig. S1 and Table S3). Within the fibrocystin family, the region was named the C6 cassette since it contains 6 highly conserved cysteines that form stabilizing disulfide linkages downstream of the PA14 domain.

**Fig. 2.**
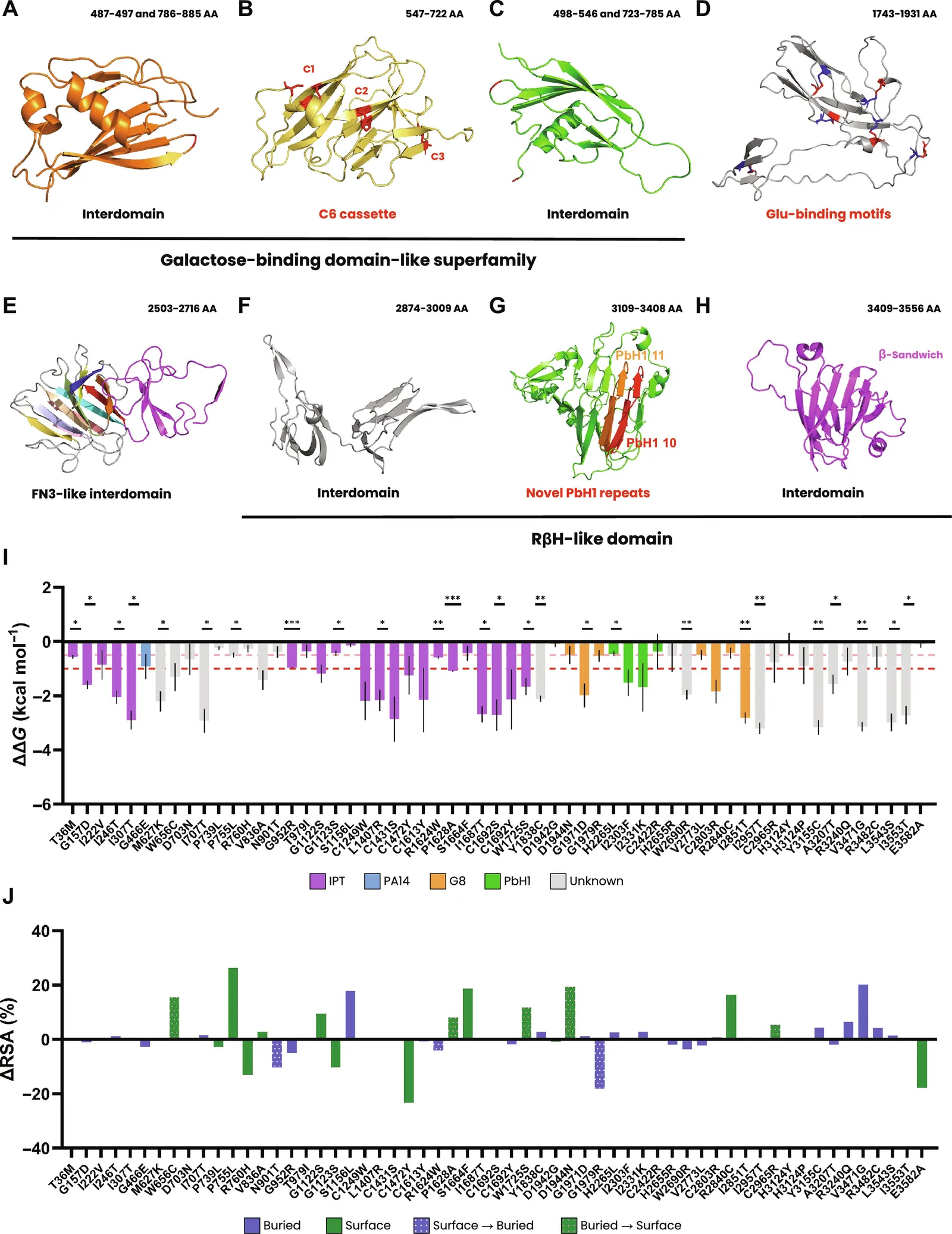
Molecular basis for interdomains of fibrocystin and broad structural trends among pathogenic *PKHD1* variants. The interdomain after PA14 and before IPT 4 is divided into 3 domains including (A) immunoglobulin-like fold domain, (B) C6 cassette, and (C) β-domain. From FoldSeek homology analyses, this region has been proposed as the galactose-binding domain. (D) The Glu-binding motif occurs after IPT 12 and before the G8 1 domain composed of 6 disulfide bonds stabilizing the domain. (E) A 6:5 β-sandwich is formed as the interdomain after PbH1 6 and before G8 2 domains. (F) The unknown interdomains after the PbH1 repeats and before the SEA domain is composed of (G) PbH1 10/11 repeats and (H) β-sandwich. This region has been suggested to be a RβH-like domain. (I) The change in free energy upon point mutation has been calculated for pathogenic ClinVar *PKHD1* variants implicated in autosomal recessive polycystic kidney disease (ARPKD). Bars are represented by the mean and error bars as the standard error of the mean (*n* = 3). Statistical testing was determined by a one-sample, Student *t* test. The threshold at −0.5 kcal mol^−1^ indicates that a variant is weakly destabilizing and −1 kcal mol^−1^ as severely destabilizing, respectively. (J) Likewise, the change in relative solvent accessibility (RSA) of the pathogenic ClinVar variants is displayed. RβH, single-stranded right-handed beta-helix, pectin lyase-like domain.

Furthermore, we assessed the interdomain between IPT 12 and the first G8 domain from residues 1743 and 1931. The homologs were unremarkable from those found for the C6 cassette, but we identified a highly conserved sequence among the fibrocystin family including E-X-E binding motifs closely upstream of G8. These sites include residues 1805–1807, 1871–1873, 1887–1889, 1889–1891, 1891–1893, and 1893–1895. As these residues are found on the surface of our structural model, we suspected that protein–protein interactions may be mediated at these sites. We also identified 6 conserved disulfide linkages buried in the structure (Fig. 2D). The interdomain at position 2503 to 2716 after PbH1 6 and before G8 2 forms a β-sandwich composed of 6 by 5 β-strands on both sides (Fig. 2E). Although prior works have postulated a homology of *CEMIP2* and *PKHDL1*, our homolog analyses were unremarkable [23]. Likewise, the interdomain occurring at position 2874 to 3009 that is between PbH1 8 and the G8 unit does not form an individual domain.

Interestingly, when mapping position 3109 to 3408 onto the AF3 model, there was a striking resemblance to PbH1 repeats (Fig. 2F). The original description of fibrocystin confidently identified 9 PbH1 repeats but did not exclude the possibility of additional repeats downstream [5]. Based on sequence and the structural model, we have identified 2 additional repeats, where PbH1 10 and PbH1 11 occur from residues 3141–3162 and 3163–3184, respectively. The remaining elements were unremarkable in the region and the downstream residues from 3409–3556 formed a β-sandwich. Overall, our analyses have refined the molecular architecture of fibrocystin.

### *PKHD1* missense variants often cause buried, loss-of-function mechanisms in fibrocystin, leading to ARPKD

Using our refined molecular model of fibrocystin, we sought to predict a molecular basis for disease-causing missense variants leading to ARPKD. From ClinVar, we identified 59 pathogenic missense variants occurring on high pLDDT residues of the predicted structure and subsequently performed biophysical studies. In Fig. 2I, we estimated the change in free energy of folding upon amino acid substitution (ΔΔ*G*) for all variants. We placed thresholds at −0.5 and −1 kcal mol^−1^ as indicators of weak and strong destabilizing effects, respectively. Among all variants, 52.54% (31/59) (range: −1.08, −3.21 kcal mol^−1^) had an average ΔΔ*G* beyond the strong destabilizing threshold while 69.49% (41/59) (−0.52, −3.21 kcal mol^−1^) were above the weakly destabilizing threshold. Only 3.39% (2/59) (−0.07, −0.11 kcal mol^−1^) of variants were within the error of a stabilizing effect on the structure. In Fig. 2J, we estimated the change in relative solvent accessibility (RSA) as a determinant of whether the mutant residue is either buried or on its surface. Of all variants, 77.96% (46/59) occurred on buried, wild-type residues and 3.39% (2/59) were initially on the surface and were subsequently buried after *in silico* mutagenesis. Likewise, we identified 22.04% (13/59) of variants that were initially surface exposed. Therefore, our data suggest that disease-causing missense variants often localize in buried regions and may destabilize fibrocystin.

Next, we performed case studies of essential *PKHD1* variants, which includes p.T36M (c.107C>T), p.I307T (c.920T>C), p.C1431S (c.4292G>C), p.Y1838C (c.5513A>G), p.A3207T (9619G>A), and p.V3471G (c.10412T>G).

First, we evaluated the p.T36M missense variant which is the most frequent variant accounting for 21% of cases and associated with a severe form of ARPKD [24]. The p.T36M variant is buried in the IPT 1 domain and causes significantly weak destabilization to the structure by −0.560 ± 0.060 kcal mol^−1^ (*P* = 0.0116) (Fig. 3A). In Fig. 3B, we found that Thr36 is involved in an extensive hydrogen bond network by the side-chain hydroxyl with Ser31, Thr93, and Lys234. The side-chain amine of Thr36 is also involved in an interaction with the side-chain hydroxyl of Thr93. However, after mutagenesis, the majority of this bond network is lost and 2 interactions are retained with the hydroxyl of Thr93 (Fig. 3C). Although p.T36M is buried in IPT 1 and causes destabilization of protein folding, it may not be its disease-causing mechanism as prior studies have speculated that the variant disrupts the signal peptide sequence, leading to protein aggregation and failure to localize in the cell membrane [24].

**Fig. 3.**
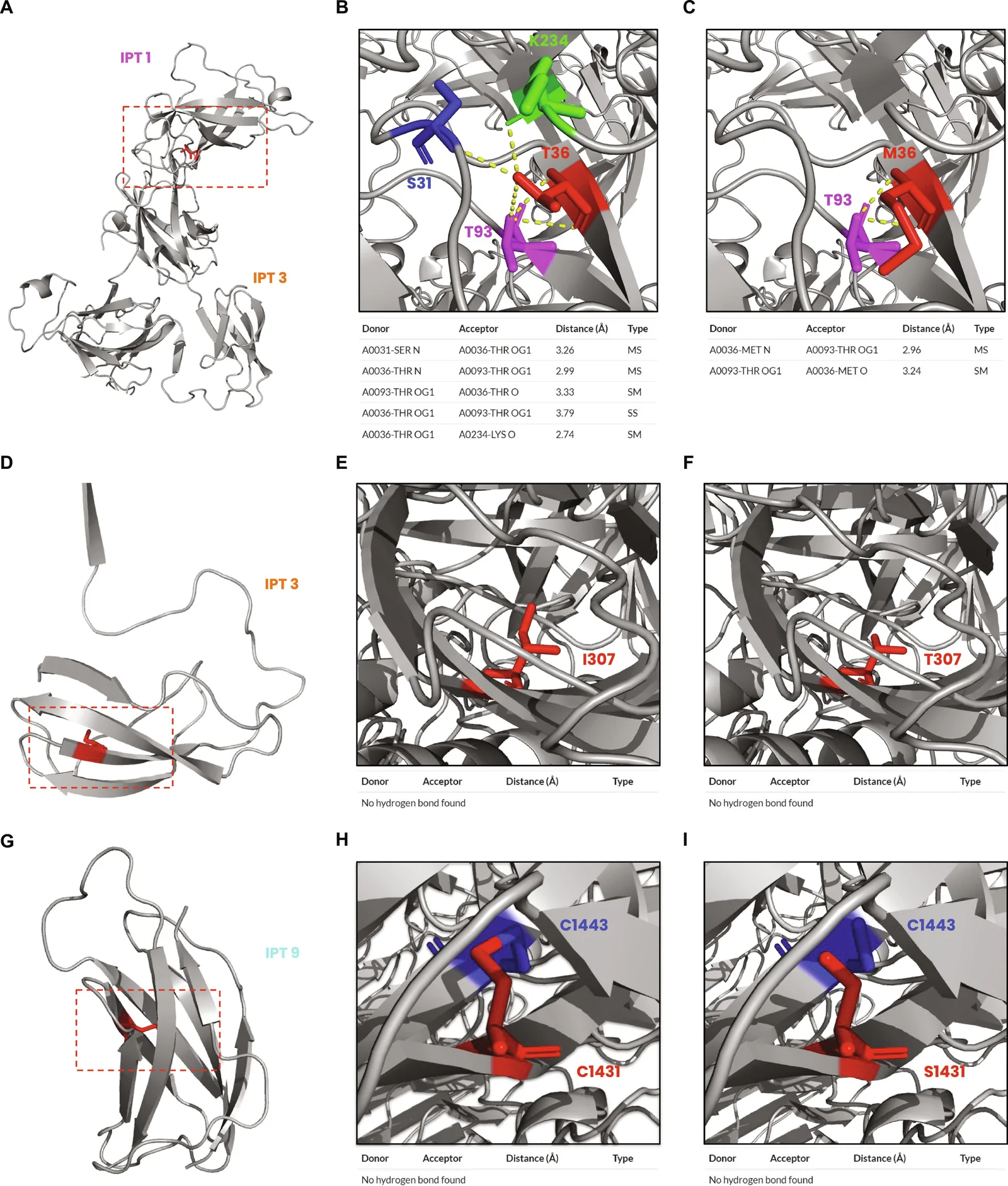
Molecular basis of influential *PKHD1* variants involved in ARPKD. (A) T36M, the most common variant found in patients with ARPKD, is involved in interactions within IPT 1 and 3. (B) Thr36 is involved in a hydrogen bond network with Ser31, Thr93, and Lys234, (C) which is broken upon mutagenesis and only retains interactions with Thr93. (D) Ile307 is buried in the IPT 3 domain. (E) Ile307 is hydrophobic and not involved in any polar interactions, (F) and upon mutagenesis is hydrophilic but still not involved in interactions. (G) Likewise, Cys1443 is buried in the inner folding of IPT 9, and (H) is involved in a disulfide bond with Cys1431. (I) However, upon mutagenesis, the disulfide bond is broken and the structure is destabilized. MM, main chain to main chain; MS, main chain to side chain; SS, side chain to side chain

Next, we analyzed the p.I307T missense variant, as it is the most disruptive variant in the IPT domains. Variant p.I307T is buried in the IPT 3 domain and leads to significant destabilization of fibrocystin by −2.893 ± 0.344 kcal mol^−1^ (*P* = 0.0139). I307 is not involved in interactions and introduces a hydrophilic residue (Fig. 3D to F). Likewise, we investigated the p.C1431S variant found on IPT 9 (Fig. 3G to I) because our supplemental analysis (Fig. S2) demonstrates that the disulfide linkages stabilize the IPT domains. Cys1431 is buried in the structure and involved in a single disulfide bond with C1443. Upon mutagenesis, this interaction is broken and leads to destabilizing effects on the structure of −2.863 ± 0.883 kcal mol^−1^, though not significant (*P* = 0.0753).

Our analyses continued to p.Y1838C, which has been suggested to be a founder variant in Turkish populations [3]. In Fig. 4A, we found that Tyr1838 was buried in the structure of the Glu-binding motifs. In the wild-type, the Tyr1838 side-chain hydroxyl was involved in a single hydrogen bond interaction with the main-chain carbonyl of Leu1790 (Fig. 4B). However, upon mutagenesis, this bond is broken and results in significant destabilization by −2.110 ± 0.110 kcal mol^−1^ (*P* = 0.0027) (Fig. 4C). The p.A3207T variant is buried in the region after PbH1 11 (Fig. 4D), which has had consistent non-perinatal disease onset among patients with ARPKD [25]. We found that the carbonyl side chain of Ala3207 was involved in a single hydrogen bond with the main-chain amine of Thr3207, but after mutagenesis it was no longer involved in any interactions (Fig. 4E and F). This substitution did lead to destabilization by −1.570 ± 0.360 kcal mol^−1^ (*P* = 0.0448).

**Fig. 4.**
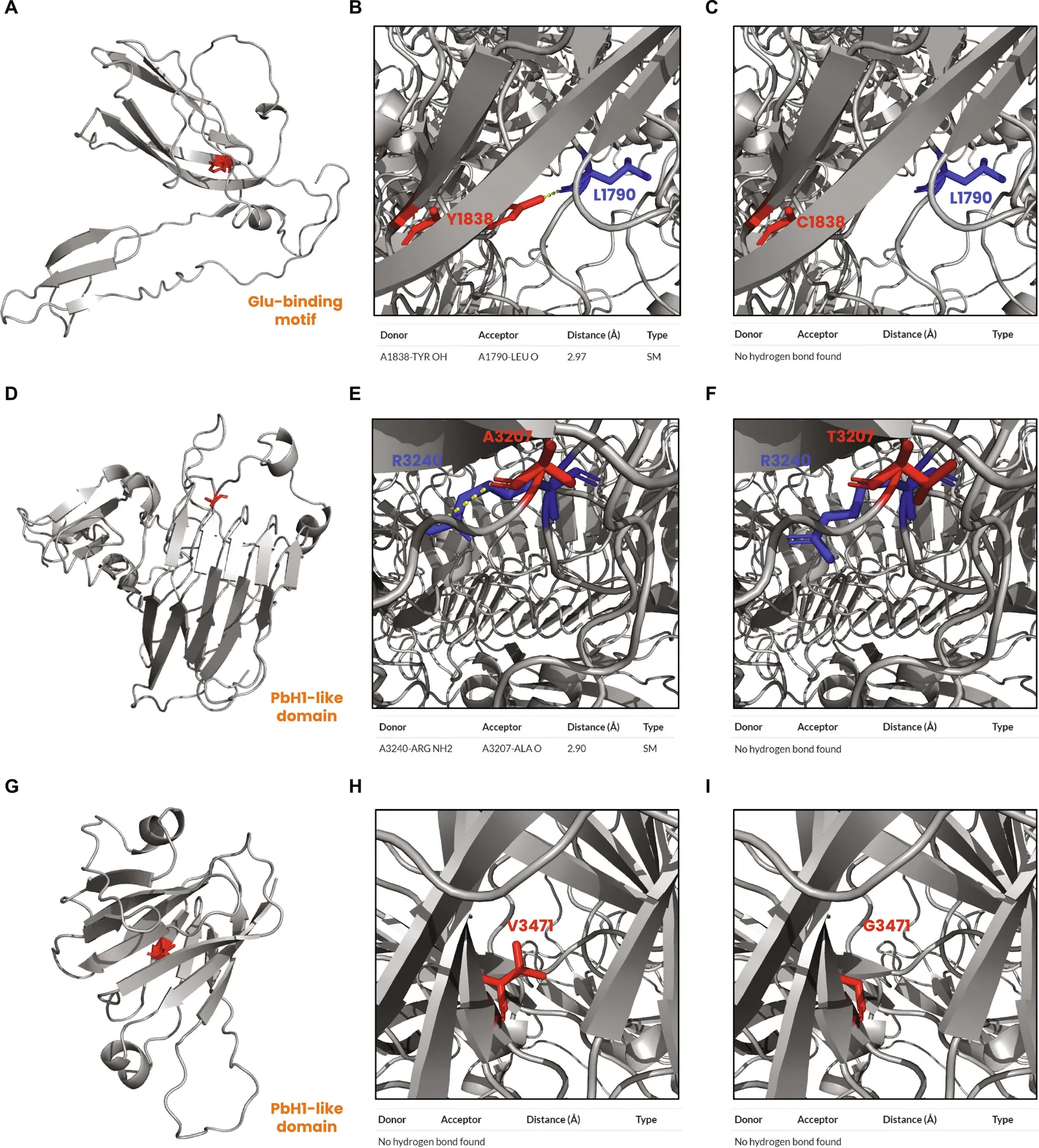
Molecular basis of p.Y1838C, p.A3207T, and p.V3471G ARPKD variants. (A) p.Y1838C is buried in the Glu-binding motifs, (B) where Tyr1838 is involved in a single hydrogen bond interaction with Leu1790, (C) but upon mutagenesis appears to be broken. (D) A3207T is located in the PbH1-like domain, (E) and is involved in a single hydrogen bond interaction with Arg3240, (F) which is lost after mutagenesis. (G) p.V3471G is also buried in the fold of the PbH1-like domain, (H) and is not involved in any interaction. (I) This remains similar after mutagenesis.

Our biophysical analyses concluded with p.V3471G which has been linked to early kidney failure among patients with ARPKD [3]. This variant is found in the interdomain buried within the β-sandwich. Both the wild-type and mutant residue were not involved in any other interactions (Fig. 4G and H) and resulted in severe destabilizing effects of −3.140 ± 0.175 kcal mol^−1^ (*P* = 0.0031) onto the structure (Fig. 4I).

Lastly, as a negative control, we performed the same analyses on 30 benign variants from ClinVar. In Fig. S3A, 96.66% (29/30) (−1.47, 0.36 kcal mol^−1^) of benign *PKHD1* variants did not impose destabilizing or stabilizing effects and 75% (25/30) were found on the surface of fibrocystin. Only 20% (6/30) of benign variants were found on structural domains, including IPT and G8. We consistently show that benign variants are surface exposed (Fig. S3B). In Fig. S4A and B, using a statistical approach instead of prediction, we observed the same correlation among benign and pathogenic variants. Overall, from our structural framework, disease-causing missense variants implicated in ARPKD tend to be buried and impose loss-of-function effects (78%).

### The first idealized molecular model of fibrocystin

After confirming the existing domain architecture of fibrocystin, describing new nomenclature for unresolved regions, and explaining the predicted molecular basis of disease-causing variants, our final objective for the structural framework was to obtain an idealized biophysical model of fibrocystin. Although the annotated domains are highly confident, the intrinsically disordered regions create a tangled architecture that prevents its serpentine curvature. Here, we preserved the atomic coordinates for each secondary structural element while altering the trajectories of the linker regions to create a more idealized model of fibrocystin (“Materials and Methods” section). In Fig. 5, we have successfully created the first idealized, structural model of fibrocystin labeled from its domain annotations, pLDDT, and electrostatic surfaces. The dimensions (height × width × depth) of our model are 108 nm × 36 nm × 8 nm. Our molecular model is the closest representation of fibrocystin without experimental resolution and has potential to aid in visualizing genetic variants, improving diagnostic methods, and explaining the genotype–phenotype correlations of *PKHD1* variants.

**Fig. 5.**
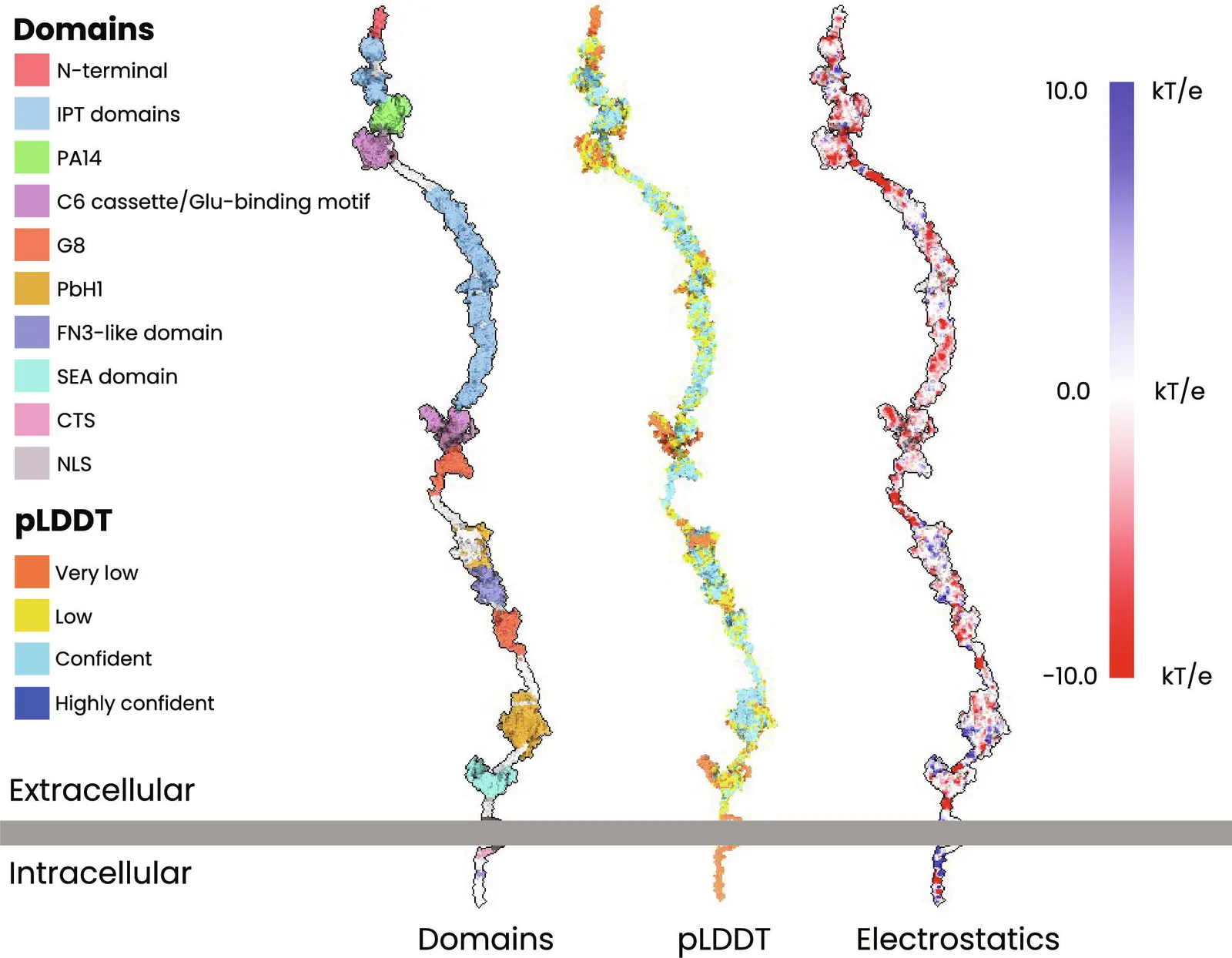
The idealized molecular model of fibrocystin. The monomer forms a serpentine-like curvature, which has been labeled according to surface electrostatics, domain annotations, and pLDDT.

### The molecular model of fibrocystin explains kidney survival and hepatic phenotypes for missense variants in amino acids 709–1837 and 1838–2624

With a defined molecular model for fibrocystin, we sought to first explain the genotype–phenotype correlations of PKHD1 variants from Burgmaier *et al*. [16], acknowledging that the correlations were underpowered. We replicated their Kaplan–Meier survival curves among the 138 patients with ARPKD stratified by their missense variants in AA1–708, 709–1837, 1838–2624, and 2625–4074 (“Materials and Methods” section). Detailed methods relating to the ARegPKD cohort, stratification methodologies, and their variables are available in Burgmaier *et al*. [16]. To identify potential covariates from the molecular model, we developed 2 Cox proportional-hazards models using RSA and ΔΔ*G* per missense substitution.

For the ΔΔ*G* covariate model (Fig. 6A), we found that for each 1 kcal mol^−1^ increase in destabilization from a missense variant, the hazards of developing kidney failure increase by 8% (hazard ratio [HR]: 0.92; 95% confidence interval [CI], 0.88 to 0.96; *P* < 0.005). This significance was driven by an 81% lower hazard of kidney failure by patients with variants at AA709–1837 (HR: 0.19; 95% CI, 0.06 to 0.58; *P* < 0.005). However, ΔΔ*G* was not a significant predictor for either portal hypertension (*P* = 0.08) or hepatic complications (*P* = 0.27). Likewise, from our RSA covariate model (Fig. 6B), we found that all individual class predictions lacked significance, but revealed that AA709–1837 lowered the hazard of kidney failure (HR: 0.20; 95% CI, 0.07 to 0.61; *P* < 0.005) and AA2625–4074 increased the risk of hepatic complications (HR: 4.39; 95% CI, 1.21 to 4.39; *P* = 0.02).

**Fig. 6.**
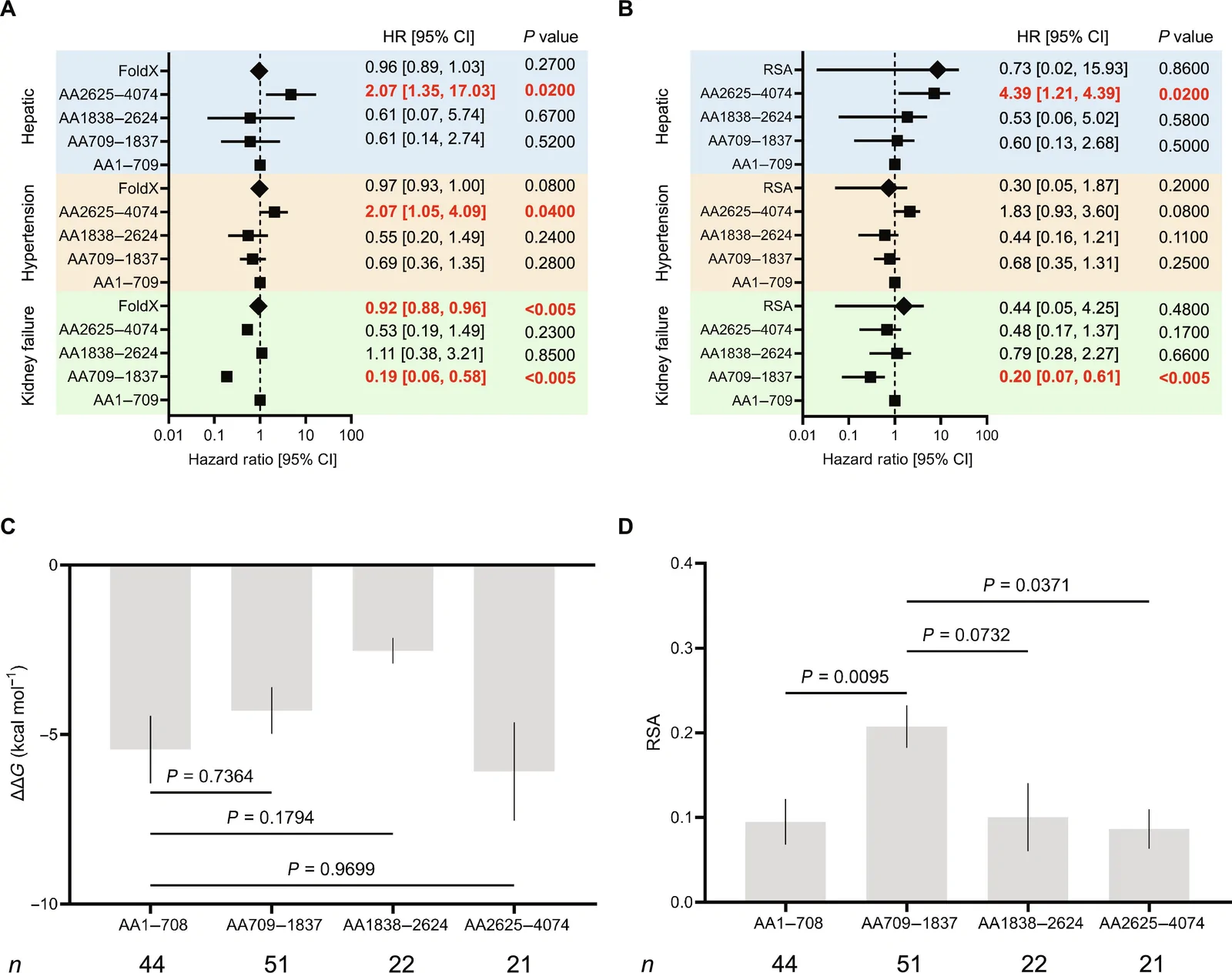
Hypotheses explaining the genotype–phenotype correlations for ARPKD. (A) Forest plot of the hazard ratio (HR) of the Cox proportional-hazards model for FoldX estimations of change in free energy (ΔΔ*G*) adjusted by functional classes of fibrocystin. CI, confidence interval. (B) Forest plot of the HR of the Cox proportional-hazards model for wild-type RSA adjusted by functional classes of fibrocystin. (C) Mean ΔΔ*G* among the functional classes of missense variants. Bars are represented by the mean and error bars as the standard error. (D) Mean wild-type RSA among all functional classes from Burgmaier *et al*. [16], where AA709–1837 has significantly greater surface exposure compared to other variants. Statistical testing was determined by a one-way analysis of variance, followed by Dunnett’s post hoc test. Sample sizes are provided in each bar.

To verify the significant correlations observed in the covariate models, we measured each covariate across each missense functional class. In Fig. 6C, we show that there are no significant differences in ΔΔ*G* among variants of all functional classes, although AA1838–2624 is least affected (*P* = 0.1219), indicating that destabilization did not distinguish patients by variant severity. Nonetheless, ΔΔ*G* retained prognostic significance in survival models, suggesting that subtle, individual-level variation in protein stability may influence disease progression even when class-level data are similar. Lastly, and most importantly, we found that variants in AA709–1837 were more surface exposed than all other *PKHD1* functional classes, though not significantly compared to AA1838–2624 (Fig. 6D). Our model thereby hypothesizes that the protective kidney survival outcomes from AA709–1837 are due to surface-exposed missense variants. Likewise, the protective hepatic phenotypes of missense variants in AA1838–2624 may be due to less severe destabilization upon substitution.

## Discussion

This study presents the first putative molecular model of fibrocystin, confirming the established structural domains, refining unresolved regions, explaining the mechanisms for *PKHD1* missense variants, and providing a hypothesis that explains protective genotype–phenotype correlations. Despite the lack of attention and priority to fibrocystin in the 24 years since its discovery, our study attempts to address this gap. Using AlphaFold as a base structural framework, we were successful in creating an idealized molecular model (Fig. 5) that aligns with the known schematics (Fig. 1A). Our work has major implications for both basic and clinical translation efforts for ARPKD. The molecular model may assist in (a) visualizing PKHD1 variants, (b) variant interpretation, and (c) prioritization of basic studies investigating different variant phenotypes. Despite recent difficulties to develop a structure of fibrocystin [26], we believe our study provides a strong, validated fibrocystin model.

One intriguing question that remains from our study is the role of domain architecture in driving the phenotypic differences observed between functional classes. AA709–1837 contains the majority of IPT domains, which are implicated in dimerization, signaling, and ligand–binding interactions, and the concentration of surface-exposed pathogenic variants within this region raises the possibility that disrupted protein–protein interactions underlie its distinct renal phenotype. The exact binding partners or ligands disrupted by these surface-exposed variants in this region remain unknown, and studies using co-immunoprecipitation or targeted mutagenesis are promising. Another lingering correlation from this study is how missense variants in AA1838–2624 are buried in the structure but do not cause significant destabilizing effects that cause protective liver, but not kidney, phenotypes.

Ultimately, this study on the structural architecture of fibrocystin contributes foundational information for ARPKD research. This study is critical because it hypothesizes a molecular mechanism explaining the genotype–phenotype correlations described in the last 5 years. Currently, there are gaps in our interpretation of genetic sequencing of *PKHD1*, and we lack U.S. Food and Drug Administration-approved disease-modifying therapies for treating ARPKD. Recent efforts for ARPKD have been focused on making risk models for kidney survival, with the notable addition of limited patient data and external validation [27,28]. Our hope is that this work will enable new directions in both basic research for fibrocystin and translational efforts for ultimately contributing to the development of specific therapies for ARPKD.

## Materials and Methods

### Study participants

For genotype–phenotype correlations, we used the cohort of 304 patients with ARPKD from Burgmaier *et al.* [16] derived from the ARegPKD registry.

### Biophysical analyses of fibrocystin

We obtained unsupervised predictions of fibrocystin monomers from AF3. *In silico* mutagenesis was performed using the SCWRL4 [29]. From the structure, we calculated the RSA of all residues excluding surface interfaces. Destabilization energies were estimated using both established statistical and machine-learning approaches for missense variants [30–33].

### Structural quality

The structure of fibrocystin was constructed using the AF3 model with provided training parameters from DeepMind [1]. Five models were ranked using the following equation: 0.8 × ipTM × 0.2 × pTM + 0.5 × disorder − 100 × clash, where ipTM is the interface predicted template modeling score and pTM is the predicted template modeling score. We compared the top prediction (model 0). The pLDDT was used for comparing both models as an internal validation metric. We showed each predicted domain of *PKHD1* as provided in UniProt and Protein Atlas.

### Genotype–phenotype correlations and statistical validity

Burgmaier *et al*. [16] created a cohort of 304 patients with ARPKD, including 138 for survival analysis stratified by patients with missense/null or missense/missense variants in 4 functional classes of fibrocystin. In Table S1, we show that these correlations should be interpreted cautiously as the statistics lack statistical power. This has been acknowledged by Burgmaier *et al.* [16], and we were therefore able to refine the observed correlations from their prior clinical investigation.

### Statistical analyses and reproducibility

All data and *P* values are indicated in the corresponding figures and tables: ns, non-significance; *P < 0.05; **P < 0.01; ***P < 0.001; ****P < 0.0001. There was neither randomization nor blinding of the investigators during both data collection and outcome assessment of this study. Kaplan–Meier survival curves paired with log-rank (Mantel–Cox) tests were applied. All descriptive statistical tests and visualizations were performed in GraphPad Prism. The source file has been provided in GitHub.

### Variant selection

After validating the predicted structure of fibrocystin, we evaluated the structural effects among pathogenic variants of *PKHD1*. Using ClinVar, we identified 1,709 missense variants, which was reduced to 66 pathogenic variants. After obtaining the pathogenic variants, we reduced our sample size to those with pLDDT >70, culminating in 59 pathogenic variants. We chose to look at residues with high pLDDT scores because it ensured high-quality structural analysis and removed start-loss variants (i.e., M1I, M1V, and M1T). We labeled the location of each residue by its predicted domain provided by UniProt (P08F94) and those not found on any domain as “Unknown”. Variants located on unknown regions that were later resolved in this study have been described. Additionally, from ClinVar, there are 30 benign variants used for analyses in this study.

### Structural analyses

*In silico* mutagenesis was performed using the established SCWRL4 model predicting side-chain conformations with high accuracy [29,34]. Clash scores of the wild-type and mutant residues were calculated by the “clashlistcluster” feature from MolProbity, which determines the overall steric overlaps between non-bonded atoms [35]. The RSA was calculated following DSSP for the entire protein, where the accessible surface area of a residue is divided by the maximum solvent accessibility outlined by Tien *et al*. and Chothia [36,37]. A residue was “buried” if the RSA was below 25% and “surface-exposed” if greater than 25%. From our structure, we did not identify any molecular interactions occurring at the interfaces of fibrocystin, and there is no existing literature supporting intramolecular interactions of individual domains.

### Nomenclature for novel interdomains

To define the nomenclature of unresolved regions of fibrocystin, we used FoldSeek and HMMER [22,27]. We narrowed our analysis to the target sequence found in the CATH50 protein structure database from University College London and UniProt, and subsequently to sequences from *Homo sapiens* and significantly low *E* values below 0.005.

### Formation of the idealized model of fibrocystin

To create the idealized serpentine architecture of fibrocystin, we used the template model and used biopython to adjust the structure. We treated the secondary structural elements as rigid bodies in the model to preserve their atomic coordinates (excluding loops). The interdomain linkers that create the AlphaFold model were “straightened” by repositioning the trajectories of the residues while maintaining steric constraints. Our model represents an idealized architecture of fibrocystin, not the native monomer.

### Protein-stabilization analysis

We followed the validated open-source protocol [28] in estimating ΔΔ*G* using 6 differing algorithmic approaches. Our method has been used in providing baseline biophysical data for significant missense variants of the triggering receptor expressed on myeloid cells 2 (*TREM2*) [38]. However, there are limitations to this approach for larger proteins as demonstrated in the *PIEZO1* (2,521 amino acids) that relied solely from a single approach [39]. We used 4 approaches to estimating the true ΔΔ*G* for the pathogenic *PKHD1* variants in this study. We intentionally used PremPS [40], a machine-learning model that was trained on a balanced dataset of stabilizing and destabilizing missense variants, along with DynaMut2 [30] and DDMUT [31] that follow deep-learning approaches. The statistical model, FoldX, was used with an established standard error of 0.85 kcal mol^−1^.

### Genotype–phenotype correlations and post hoc analyses

From the source patient data provided from Burgmaier *et al.* [16], we replicated the survival analyses performed on 138 patients with ARPKD divided by 4 functional classes (AA1–708, 709–1837, 1838–2624, and 2625–4074). After replication, we performed Cox proportional-hazards regression to determine potential covariates of the model. For each functional class, we applied one-hot encoding with one category dropped as the reference level to prevent perfect multicollinearity and to allow inclusion of the resulting indicator variables as covariates in the model. In compound heterozygotes, we took the average of the 2 missense variants for the RSA (wild-type) and ΔΔ*G* (FoldX) predictor variables because each allele and its corresponding impact is independent from one another per patient. We must denote that ΔΔ*G*_average_ may not represent the full biological reality of the patient, but it is more stringent than additive methods in calculations. Furthermore, due to the limited sample size of patients with ARPKD from Burgmaier *et al.* [16], which is further reduced at downstream time points, we chose to not refine the functional classes to the individual proposed domains from this study.

### Limitations

There are a few limitations to this study in several ways. First, our wild-type fibrocystin protein structure needs validation using traditional biophysical techniques of NMR or x-ray crystallography. Fundamentally, the computational approach used for this study is not meant to replace crystal structures, but rather to provide baseline biochemical data on a large scale of wild-type and mutant structures that would otherwise be experimentally unfeasible. Therefore, biophysical resolution of fibrocystin would only strengthen the findings being reported in this study. Another drawback lies in the *in silico* mutagenesis techniques. Within the field of protein design, current biophysical models do not consider dynamic conformational changes and are in rigid-structure predictions, even with consideration of molecular dynamic simulations. AA missense variations that affect the protein backbone are unable to be predicted. Our data are conservative based on the position and physicochemical properties of the wild-type residue and its expected alterations upon substitution. There are also 2 limitations with sample size of this study. First, due to the size of fibrocystin, we were limited in the number of computational approaches used to estimate the change in free energy upon missense substitution. To address this weakness, we also relied on the traditional FoldX statistical approach for estimation. The second is the sample size of patients with ARPKD used in Burgmaier *et al.* [16] that likely lowered the statistical power of the Cox proportional-hazards regression performed with the structural data. We also addressed this limitation by performing individual analysis of each covariate to decipher differences among the different functional classes.

## Data Availability

All supplementary data reported have been provided in the following GitHub repository: https://github.com/Joshua-Pillai/PKHD1. The wild-type protein structure of fibrocystin has been deposited in Model Archive: doi.org/10.5452/ma-0flp4.
