# Supplementary material for: Autosomal Recessive Polycystic Kidney Disease: The Complete Molecular Architecture for the PKHD1 Gene and Its Protein
Source: Comput Struct Biotechnol J. 2026 Nov 12;35(1):0261. doi: 10.34133/csbj.0261 (PMC13755561; doi:10.34133/csbj.0261)
Supplement: Supplementary file 1 [file csbj.0261.f1.docx]

**SUPPLEMENTAL MATERIAL**

**Autosomal Recessive Polycystic Kidney Disease: The complete molecular architecture for the *PKHD1* gene and its protein**

Joshua P. Pillai^1,2^, Sidhant N. Puntambekar^1,3^, Ana C. Onuchic-Whitford^3,4,5^, Frederico Ferreira^6,7^, Matthew G. Sampson^1,2,3,4,5+^, Luiz F. Onuchic^6,7^, John A. Sayer^8,9,10+^

^1^Division of Nephrology, Boston Children’s Hospital, Boston, MA, USA.

^2^Department of Pediatrics, Harvard Medical School, Boston, MA, USA.

^3^Kidney Disease Initiative, Broad Institute, Cambridge, MA, USA.

^4^Department of Pediatrics, Harvard Medical School, Boston, MA, USA.

^5^Division of Renal Medicine, Brigham and Women’s Hospital, Boston, MA, USA.

^6^Division of Nephrology, School of Medicine, University of São Paulo, São Paulo, Brazil.

^7^Division of Molecular Medicine, School of Medicine, University of São Paulo, São Paulo, Brazil.

^8^Newcastle University, Newcastle Upon Tyne, UK.

^9^Newcastle Hospitals NHS Foundation Trust, Newcastle Upon Tyne, UK.

^10^NIHR Newcastle Biomedical Research Centre, Newcastle upon Tyne, UK.

+ these authors jointly supervised the work

Correspondence to:

John A. Sayer - john.sayer@newcastle.ac.uk

**Supplemental Figure Description**

**Figure S1. Sequence analysis of protein homologs for the C6 cassette.** The distance of the conserved sequence from the established PA14 is shown in the figure.

**Figure S2. Cysteines in the complete fibrocystin structure. (a)** Distribution of cysteines across all domains, where there appears to be highest concentrations in the CRD1 and CRD2 domain. **(b)** Disulfide bonds appear in IPT 1 and IPT 3, but not IPT 2 or the PA14 domain. **(c)** Similarly, disulfide bonds appear in IPT 4, IPT 5, and two within IPT 7. **(d)** A single disulfide bond appears present in the IPT 8 to IPT 12 subdomain.

**Figure S3. Free energy change of benign *PKHD1* variants. (a)** Average free energy change of benign variants calculated using ML-based approaches, where there was no statistical significance from 0 kcal/mol^-1^. Statistical testing was determined by a one-sample, Student’s *t*-test. **(b)** The change in RSA among all ClinVar benign variants. 25 variants are located on the surface, and an additional variant changes from being buried to on the surface (p.D2433V).

**Figure S4. Validation of free energy changes of both pathogenic and benign *PKHD1* variants from FoldX. (a)** 67.79% (40 / 59) of pathogenic variants had a destabilization beyond -2.5 kcal/mol^-1^ and 32.20% (19 / 59) were highly destabilizing beyond -5 kcal/mol^-1^. **(b)** Only 13.33% (4 / 30) of benign variants were beyond the weakly destabilizing threshold and 0% (0 / 30) for the severe destabilization. The overall trends observed from FoldX statistical predictions validate previous data.

**Table S1. Bonferroni correction of Burgmaier et al., (2021) genotype-phenotype correlations.** Most p-values are non-significant upon correction.

**Table S2. Internal validation metrics of the AF3 model predictions of fibrocystin.** Reproducibility code has been provided in the GitHub repository.

**Table S3. Sequence analysis of C6 cassette downstream of PA14.** See table and sequence below for precise cysteines forming disulfide linkages.

**Table S4. PAE for each domain and interdomain of fibrocystin model.**

Table S1. Pillai et al., (2026).

| **Comparison** | ***n* (709-1837/other)** | **Fisher’s *p*** | **Bonferroni correction** |
| --- | --- | --- | --- |
| AA1-708 | 51/44 | 0.0009 | 0.0027 |
| AA1838-2624 | 51/22 | **0.1175** | **0.3525** |
| AA2625-4074 | 51/21 | **0.0541** | **0.1623** |
| **Comparison** | ***n* (1838-2624/other)** | **Fisher’s *p*** | **Bonferroni correction** |
| AA1-708 | 22/44 | **0.1066** | **0.3198** |
| AA709-1837 | 22/51 | **0.4179** | **0.9999** |
| AA2625-4074 | 22/21 | 0.0007 | 0.0021 |
| **Comparison** | ***n* (1838-2624/other)** | **Fisher’s *p*** | **Bonferroni correction** |
| AA1-708 | 22/44 | **0.6549** | **0.9999** |
| AA709-1837 | 22/51 | **0.9999** | **0.9999** |
| AA2625-4074 | 22/21 | 0.0093 | 0.0280 |
| **Comparison** | ***n* (2625-4074/other)** | **Fisher’s *p*** | **Bonferroni correction** |
| AA1-708 | 21/44 | **0.5873** | **0.9999** |
| AA709-1837 | 21/51 | **0.0541** | **0.1624** |
| AA1838-2624 | 21/22 | **0.7360** | **0.9999** |
| **Comparison** | ***n* (2625-4074/other)** | **Fisher’s *p*** | **Bonferroni correction** |
| AA1-708 | 21/44 | 0.0194 | **0.0581** |
| AA709-1837 | 21/51 | 0.0015 | 0.0046 |
| **Comparison** | ***n* (2625-4074/other)** | **Fisher’s *p*** | **Bonforroni correction** |
| AA1-708 | 21/44 | 0.0319 | **0.0957** |
| AA709-1837 | 21/51 | 0.0015 | 0.0045 |
| AA1838-2624 | 21/22 | 0.0007 | 0.0021 |

Table S2. Pillai et al., (2026).

| **Model** | **iptm** | **pae_min** | **chain_ptm** | **disordered** | **clash** | **rank** | **pLDDT** |
| --- | --- | --- | --- | --- | --- | --- | --- |
| **0** | 0.51 | 0.76 | 0.51 | 0.09 | 0.0 | 0.56 | 73.59 |
| **1** | 0.52 | 0.76 | 0.52 | 0.08 | 0.0 | 0.56 | 73.68 |
| **2** | 0.51 | 0.76 | 0.51 | 0.09 | 0.0 | 0.55 | 73.50 |
| **3** | 0.51 | 0.76 | 0.51 | 0.08 | 0.0 | 0.55 | 73.58 |
| **4** | 0.51 | 0.76 | 0.51 | 0.08 | 0.0 | 0.55 | 73.42 |

Table S3. Pillai et al., (2026).

| **HMMER** | **Species** | **HMMER name** |
| --- | --- | --- |
| P08F94 | *Homo sapiens* | **Fibrocystin** |
| A0A7J7FP51_DICBM | *Diceros bicornis* | PA14 domain-containing protein (Fragment) |
| A0A4W2CG52_BOBOX | *Bos indicus × Bos taurus* | Isoform of A0A4W2BY62, PA14 domain-containing protein |
| G3T9X7_LOXAF | *Loxodonta africana* | PA14 domain-containing protein |
| A0A5N3W0F2_MUNRE | *Muntiacus reevesi* | PA14 domain-containing protein |
| A0A811YN62_NYCPR | *Nyctereutes procyonoides* | (raccoon dog) hypothetical protein |
| A0A8C0KK16_CANLU | *Canis lupus* | PA14 domain-containing protein |
| A0A5E4DBM8_MARMO | *Marmota monax* | PA14 domain-containing protein (Fragment) |
| A0A452HNI9_9SAUR | *Gopherus agassizii* | Uncharacterized protein |
| A0A8C9EYI7_PAVCR | *Pavo cristatus* | PA14 domain-containing protein |
| A0A3L8SZE5_CHLGU | *Chloebia gouldiae* | PA14 domain-containing protein |
| A0A8C3MZI7_GEOPR | *Camarhynchus parvulus* | Uncharacterized protein |
| A0A4D9ER07_9SAUR | *Platysternon megacephalum* | Ephrin type-A receptor 6 |
| A0A8B9IFM4_ANSCY | *Anser cygnoides* | PA14 domain-containing protein |
| A0A8J7NMF7_ATRSP | *Atractosteus spatula* | PKHL1 protein (Fragment) |
| A0A3S1HUE2_ELYCH | *Elysia chlorotica* | PA14 domain-containing protein (Fragment) |
| F7C2F0_MONDO | *Monodelphis domestica* | ENY2 transcription and export complex 2 subunit |
| A0A7I8VIY9_9ANNE | *Dimorphilus gyrociliatus* | DgyrCDS4618 |
| A0AAD8YR62_9TELE | *Electrophorus voltai* | PA14 domain-containing protein |

Within the C6 domain, there are four highly conserved sequences:

C547: EELLAVKCKLEPLWS

C583: TSGTEPFCGRFSLRQ

C614: LDQYTHLCLAYKGHM

C644: NMVKNTTCDWSLTRT

C660: PESWQFDCTDLWETC

C667: CTDLWETCVRCFGDL

**Disulfide Linkages:** C547-C583 (C1), C614-C660 (C2), C644-C667 (C3).

Table S4. Pillai et al., (2026).

| **Region** | **Type** | **Median PAE (Å)** | **Mean PAE (Å)** |
| --- | --- | --- | --- |
| 24-111 | Domain | 4.8 | 6.3 |
| 112-136 | Interdomain | - | - |
| 137-232 | Domain | 3.5 | 4.1 |
| 233-258 | Interdomain | - | - |
| 259-335 | Domain | 2.9 | 3.3 |
| 322-485 | Domain | 4.1 | 5.3 |
| 486-930 | Interdomain | - | - |
| 931-1015 | Domain | 3.6 | 4.1 |
| 1016-1018 | Interdomain | - | - |
| 1019-1103 | Domain | 4.3 | 4.6 |
| 1104-1107 | Interdomain | - | - |
| 1108-1192 | Domain | 4.1 | 4.5 |
| 1193-1195 | Interdomain | - | - |
| 1196-1289 | Domain | 5.4 | 6.8 |
| 1290-1300 | Interdomain | - | - |
| 1301-1382 | Domain | 4.8 | 6 |
| 1383-1388 | Interdomain | - | - |
| 1389-1481 | Domain | 3.9 | 4.6 |
| 1482-1485 | Interdomain | - | - |
| 1486-1570 | Domain | 3.7 | 4.2 |
| 1571-1572 | Interdomain | - | - |
| 1573-1659 | Domain | 3.5 | 3.9 |
| 1658-1742 | Domain | 4.2 | 5.3 |
| 1743-1931 | Interdomain | - | - |
| 1932-2053 | Domain | 2.9 | 3.7 |
| 2054-2225 | Interdomain | - | - |
| 2226-2248 | Domain | 2.2 | 2.4 |
| 2249-2271 | Domain | 2 | 2.3 |
| 2272-2291 | Interdomain | - | - |
| 2292-2325 | Domain | 2.8 | 3.8 |
| 2326-2347 | Domain | 1.8 | 2 |
| 2348-2408 | Interdomain | - | - |
| 2409-2431 | Domain | 2 | 2.1 |
| 2432-2468 | Interdomain | - | - |
| 2469-2502 | Domain | 2.6 | 2.9 |
| 2503-2746 | Interdomain | - | - |
| 2747-2873 | Domain | 3.2 | 3.8 |
| 2874-3009 | Interdomain | - | - |
| 3010-3032 | Domain | 2.1 | 2.6 |
| 3033-3055 | Domain | 1.8 | 2.1 |
| 3056-3085 | Interdomain | - | - |
| 3086-3108 | Domain | 2 | 2.2 |
| 3109-3556 | Interdomain | - | - |
| 3557-3717 | Domain | 8.3 | 11.4 |
| 3718-3858 | Interdomain | - | - |
| 3859-3881 | Domain | 22.8 | 20.1 |
| 3876-3893 | Domain | 15.9 | 15.9 |
| 3894-3951 | Interdomain | - | - |
| 3952-3976 | Domain | 18.3 | 16.2 |

Figure S1. Pillai et al., (2026).

Figure S2. Pillai et al., (2026).

Figure S3. Pillai et al., (2026).

Figure S4. Pillai et al., (2026).
